# PAIR Comparison between Two Within-Group Conditions of Resting-State fMRI Improves Classification Accuracy

**DOI:** 10.3389/fnins.2017.00740

**Published:** 2018-01-09

**Authors:** Zhen Zhou, Jian-Bao Wang, Yu-Feng Zang, Gang Pan

**Affiliations:** ^1^College of Computer Science and Technology, Zhejiang University, Hangzhou, China; ^2^Center for Cognition and Brain Disorders and the Affiliated Hospital, Hangzhou Normal University, Hangzhou, China; ^3^Zhejiang Key Laboratory for Research in Assessment of Cognitive Impairments, Hangzhou, China; ^4^Institutes of Psychological Sciences, Hangzhou Normal University, Hangzhou, China

**Keywords:** resting-state fMRI, within-group design, amplitude of low-frequency fluctuation, linear support vector machine, dimensionality reduction

## Abstract

Classification approaches have been increasingly applied to differentiate patients and normal controls using resting-state functional magnetic resonance imaging data (RS-fMRI). Although most previous classification studies have reported promising accuracy within individual datasets, achieving high levels of accuracy with multiple datasets remains challenging for two main reasons: high dimensionality, and high variability across subjects. We used two independent RS-fMRI datasets (*n* = 31, 46, respectively) both with eyes closed (EC) and eyes open (EO) conditions. For each dataset, we first reduced the number of features to a small number of brain regions with paired *t*-tests, using the amplitude of low frequency fluctuation (ALFF) as a metric. Second, we employed a new method for feature extraction, named the PAIR method, examining EC and EO as paired conditions rather than independent conditions. Specifically, for each dataset, we obtained EC minus EO (EC—EO) maps of ALFF from half of subjects (*n* = 15 for dataset-1, *n* = 23 for dataset-2) and obtained EO—EC maps from the other half (*n* = 16 for dataset-1, *n* = 23 for dataset-2). A support vector machine (SVM) method was used for classification of EC RS-fMRI mapping and EO mapping. The mean classification accuracy of the PAIR method was 91.40% for dataset-1, and 92.75% for dataset-2 in the conventional frequency band of 0.01–0.08 Hz. For cross-dataset validation, we applied the classifier from dataset-1 directly to dataset-2, and vice versa. The mean accuracy of cross-dataset validation was 94.93% for dataset-1 to dataset-2 and 90.32% for dataset-2 to dataset-1 in the 0.01–0.08 Hz range. For the UNPAIR method, classification accuracy was substantially lower (mean 69.89% for dataset-1 and 82.97% for dataset-2), and was much lower for cross-dataset validation (64.69% for dataset-1 to dataset-2 and 64.98% for dataset-2 to dataset-1) in the 0.01–0.08 Hz range. In conclusion, for within-group design studies (e.g., paired conditions or follow-up studies), we recommend the PAIR method for feature extraction. In addition, dimensionality reduction with strong prior knowledge of specific brain regions should also be considered for feature selection in neuroimaging studies.

## 1. Introduction

Blood oxygenation level dependent (BOLD) functional magnetic resonance imaging (fMRI) is a noninvasive neuroimaging technology. BOLD fMRI is extensively used because of its accessibility, ease of operation, and relatively good temporal as well as spatial resolution. Resting-state fMRI (RS-fMRI) has been increasingly utilized to evaluate the abnormal spontaneous brain activity following a seminal study by Biswal et al. (1995). To verify differences between patients and healthy controls, the univariate approach is the most widely used statistical method (i.e., comparing differences in a voxel-wise or region-wise manner). Multivariate analyses using machine learning approaches (e.g., support vector machines; SVM), can increase sensitivity. These methods have been increasingly applied as computation capacity increases (Haxby et al., 2001; Norman et al., 2006; Yang et al., 2007; Dosenbach et al., 2010; Misaki et al., 2010; Mahmoudi et al., 2012; Zhang et al., 2012; Wee et al., 2013, 2014).

A major challenge for machine learning in neuroimaging studies, including RS-fMRI, is the “curse of dimensionality,” which arises in situations involving tens of thousands of voxels but a relatively small sample size (usually < 100 subjects). As a result, classification accuracy often fails to generalize to new data. A popular method for dimensionality reduction involving conducting *t*-tests in a voxel-wise manner and selecting voxels with larger absolute *t*-values (i.e., showing a significant difference between two groups; Zhu et al., 2008; Zou et al., 2015; Liu et al., 2017). However, classification based on the features selected by *t*-test in the same dataset represents a type of circular analysis, or “double dipping” (Kriegeskorte et al., 2009). A reasonable validation procedure is to apply spatial information (i.e., the location of the voxels) as well as the classifier directly into an independent dataset. Unfortunately, few studies have employed this method. Thus, the extent to which the *t*-value of each region or voxel is correlated with the weight or importance of the feature of each region or voxel in classification accuracy is currently unclear.

Most previous classification studies have been conducted to differentiate two or more independent groups (e.g., a patient group vs. a control group). However, some experiments have used within-group designs (e.g., two conditions within the same group of subjects, or follow-up studies). For univariate statistical analysis, this within-group design must be taken into account by using, e.g., paired *t*-tests rather than independent two-sample *t*-tests. For example, in RS-fMRI studies, eyes closed (EC) and eyes open (EO) are two typical resting conditions. Although there is not a clear consensus regarding which condition should be used for clinical studies, a number of studies have confirmed that the two conditions differ significantly (Liu et al., 2013; Xu et al., 2014; Zou et al., 2015) using paired *t*-tests. Some researchers have also performed classification analysis to differentiate these two conditions (Liang et al., 2014; Zhang et al., 2015). However, these studies have typically considered EC and EO to be two independent conditions. One of these studies obtained about 75% accuracy using a stringent split-half validation procedure (Liang et al., 2014) and the other achieved 97% accuracy using leave-one-out validation (Zhang et al., 2015). Thus, it remains unclear whether the classification analysis could be performed using a paired design, rather than considering EC and EO as independent conditions.

The current study aimed to differentiate EC and EO RS-fMRI conditions using a paired design (hereafter referred to as the PAIR method) for the following considerations. First, EC and EO are two distinct RS-fMRI conditions. Second, a few previous EC vs. EO RS-fMRI studies (Yan et al., 2009; Liu et al., 2013; Yuan et al., 2014; Zou et al., 2015) have used the same analytic method, i.e., ALFF, and consistently found difference in the sensorimotor cortex, the primary auditory cortex, and the visual areas. It means that it is well known which specific brain region should contribute to the difference between EC and EO. We therefore utilized ALFF as an RS-fMRI metric. We used paired *t*-tests and selected voxels with larger absolute *t*-values for dimensionality reduction. Further, we investigated correlations between *t*-values and the weight of features in the classifier. Importantly, to test classification accuracy, we performed cross-validation on two independent datasets acquired at two research centers.

## 2. Materials and methods

### 2.1. Subjects

For cross-validation, we used two independent datasets. Dataset-1 included 34 college students (aged 19–31 years; 16 females). The data from three participants were excluded due to the low quality of spatial normalization. Thus, 31 participants were included in the final analysis. None of the participants had a history of neurological or psychiatric disorders. The study design for dataset-1 was approved by the ethics committee of the Center for Cognition and Brain Disorders, Hangzhou Normal University. Dataset-2 was obtained from a shared data source (Liu et al., 2013) and was downloaded from http://fcon_1000.projects.nitrc.org/indi/IndiPro.html (Beijing: Eyes Open Eyes Closed Study). Dataset-2 included 48 college students (aged 19–31 years; 22 females). The data from two participants were discarded due to low quality of spatial normalization. Thus, 46 participants were included in the final analysis. None of these subjects had a history of neurological or psychiatric disorders. The study design for dataset-2 was approved by the ethics committee of the Institutional Review Board of Beijing Normal University Imaging Center for Brain Research. Written informed consent was obtained from each participant before scanning.

### 2.2. MRI scanning

In each dataset, participants underwent two RS-fMRI scanning sessions (i.e., EO without fixation and EC), each lasting for 8 min. The order of the two sessions was counterbalanced across participants. The participants lay supine with their heads snugly fixed by straps and foam pads to minimize head movement. During scanning, participants were asked to lie quietly in the scanner, not to think about anything particular, and not to fall asleep. Immediately after each scanning session, the experimenter asked the participants to report their wakefulness condition during scanning. All participants reported that they had not fallen asleep during the RS-fMRI scanning.

Dataset-1 was acquired using a GE healthcare MR-750 3-T scanner (GE Medical Systems, Milwaukee, WI) with an eight-channel head coil at the Center for Cognition and Brain Disorders of Hangzhou Normal University. The BOLD images were obtained using an echo-planar image sequence with the following parameters: repetition time (TR)/echo time (TE) = 2,000/30 ms, flip angle = 60°, 37 slices, thickness/gap = 3.4/0 mm, FOV = 220 × 220 mm^2^ with an in-plane resolution of 3.44 × 3.44 mm^2^. The duration of the resting-state scan was 8 min, and included 240 images. Additionally, a 3D T1-weighted magnetization-prepared rapid gradient echo (MPRAGE) image was acquired with the following parameters: 176 sagittal slices (achieve 176 slices with two slices at each end being discarded), slice thickness/gap = 1/0 mm, in-plane resolution = 250 × 250, TR = 8100 ms, TE = 3.1 ms, inversion time (TI) = 1,100 ms, flip angle = 8°, field of view (FOV) = 250 × 250 mm^2^.

Dataset-2 was acquired using a SIEMENS TRIO 3-Tesla scanner at the Beijing Normal University Imaging Center for Brain Research. The functional images were obtained using an echo-planar imaging sequence with the following parameters: 33 axial slices, thickness/gap = 3.5/0.7 mm, in-plane resolution = 64 × 64, TR = 2,000 ms, TE = 30 ms, flip angle = 90°, FOV = 200 × 200 mm^2^. Each condition consisted of 240 functional images. In addition, a 3D T1-weighted MPRAGE image was acquired with the following parameters: 128 sagittal slices, slice thickness/gap = 1.33/0 mm, in-plane resolution = 256 × 192, TR = 2,530 ms, TE = 3.39 ms, TI = 1,100 ms, flip angle = 7°, FOV = 256 × 256 mm^2^.

### 2.3. Data preprocessing

The following preprocessing steps were performed on the fMRI data using RESTplus (http://restfmri.net/forum/RESTplusV1.2) software, including: (1) discarding the first 10 volumes of functional images; (2) slice timing; (3) head motion correction; (4) spatial normalization using T1 image unified segmentation, then resampling the functional image to 3 mm isotropic voxels; (5) spatial smoothing with an isotropic Gaussian kernel with a full width half maximum (FWHM) of 6 mm; (6) removing linear trends within the time series. None of the subjects were excluded due to excessive head motion based on criteria of > 2 mm displacement or an angular rotation of > 2° in any direction.

It should be noted that, in addition to a whole brain mask, the current study also included eyeball area with consideration that the ALFF would be substantially different between EO and EC due to blinks in the EO condition. The eyeball mask was generated using the WFU PickAtlas Tool (http://www.nitrc.org/projects/wfu_pickatlas/), with radius = 20 mm, and central coordinates at x = −36, y = 60, z = −40 and x = 36, y = 60, z = −40 for left and right eyeball, respectively. This whole brain plus eyeball mask was then resliced to voxel size = 3 × 3 × 3 mm^3^.

### 2.4. ALFF calculation of different frequency bands

ALFF measures the signal fluctuations in each single time-course. ALFF is the simplest metric among the analytic methods for RS-fMRI and has been widely used in studies of brain disorders (Zang et al., 2015). The procedure for calculating ALFF has been described in previous studies (Zang et al., 2007). After removing linear trends, the time series from each voxel was converted into a frequency domain using fast Fourier transformation (FFT) and the power spectrum was obtained. Since the power of a given frequency is proportional to the square of the amplitude of this frequency component in the original time series in time domain, the power spectrum calculated by FFT was square rooted and then averaged across the low frequency band of 0.01–0.08 Hz in each voxel. Thus, this averaged square root was considered as the ALFF. For standardization purposes, we divided the ALFF by the mean ALFF of the individual whole brain (Zang et al., 2007).

Many previous studies reported that sub-frequency bands, especially frequency bands higher than the conventional 0.01–0.08 Hz, of the RS-fMRI signal have different physiological or pathological significance. For example, previous studies have reported differences in higher frequency bands between EC and EO (Yuan et al., 2014), and abnormal ALFF in higher frequency bands in patients with chronic pain (Malinen et al., 2010; Otti et al., 2013) and epilepsy (Wang et al., 2015). Thus, we also calculated the ALFF of sub-frequency bands including 0.01–0.027 Hz (Slow-5), 0.027–0.073 Hz (Slow-4), 0.073–0.198 Hz (Slow-3), and 0.198–0.25 Hz (Slow-2) (Zuo et al., 2010; Han et al., 2011; Zhang et al., 2013), a total of five frequency bands.

### 2.5. Paired *t*-tests for univariate statistical analysis for feature selection

There are tens of thousands of voxels in a 3D whole-brain model. To avoid the over-fitting problem (Guyon and Elisseeff, 2003), we employed paired *t*-tests between EC and EO to select a limited number of voxels with larger *t*-values. The paired *t*-tests were performed in five frequency bands in each dataset. A combination threshold for single voxel of *p* < 0.01 and cluster size > 1,269 mm^3^ (47 voxels) were utilized, corresponding to a corrected *p* < 0.01 based on Monte Carlo simulations to correct for multiple comparisons across the whole brain. It should be noted that there is not a clear consensus regarding to the best multiple comparison correction method. The main purpose of the above criteria for multiple comparison correction was not to reduce false positive findings for univariate statistical analysis. Instead, it was used primarily for feature selection.

After paired *t*-tests, features were extracted in three ways. The first method used the ALFF value of peak voxels selected from paired-t maps in each frequency band of each dataset (Peak-ALFF). The second method used the mean ALFF of a spherical ROI (radius = 5 mm, totally 19 voxels) centered at the peak voxel (Mean-ALFF). The third method used the ALFF value of all voxels in the spherical ROI (All-ALFF). The number or dimension of features differed between frequency bands and datasets because the results of the paired *t*-tests were different. Here we used **D** to denote a dataset in our study, e.g., dataset-1. Thus, **D** has *m* subjects, with each subject having two resting states, which were EC and EO, respectively. We used **C** and **O** to denote the ALFF values of all voxels in the EC and EO conditions, respectively. And **F**, e.g., **F**_*peak*_, **F**_*mean*_, and **F**_*all*_ denote the MNI coordinates obtained above using these three feature extraction methods in a given frequency band in one dataset. Given a specific frequency band, after performing paired *t*-test, we got *K* clusters with *k* peak voxels. So technically, the length of **F**_*peak*_ and **F**_*mean*_ is *k* while the length of **F**_*all*_ is *k* × 19.

### 2.6. Feature extraction

Most previous discriminative studies of MRI data have focused on differentiating two independent groups of subjects (e.g., a healthy group vs. a patient group). Conventional univariate studies typically compare the two groups using independent two-sample *t*-tests. The current study used a within-group design (i.e., EC vs. EO RS-fMRI conditions) within the same group of subjects. Conventional univariate studies compare two conditions using paired *t*-tests, to test whether the mean delta differs from zero. Previous discriminative studies of EC vs. EO data have examined the two conditions as two independent groups (Liang et al., 2014; Zhang et al., 2015), using methods like two-sample *t*-tests (namely UNPAIR method in the current study). We used the paired *t*-test method to perform classification in the PAIR method, using the ALFF difference map between EC and EO of each subject for classification, rather than using the EC map and EO map independently. Specifically, we generated two independent groups of subjects, the EC—EO group and EO—EC group, respectively, for each dataset. For example, for dataset-1, half of the subjects were randomly selected with EC minus EO of the ALFF value, and the other half with EO minus EC, hereafter referred to as the **C**−**O** and **O**−**C** group, respectively. The discriminative analysis was performed in a way named PAIR method in the current study. For a dataset with *m* subjects, we randomly generated a vector **a** of length m. The number of *a*_*i*_ equal to -1 is *m*/2 and the number of *a*_*i*_ equal to 1 is also *m*/2, 1 ≤ *i* ≤ *m*. Here we used **T** to represent the dataset after pairing group, e.g., EC—EO and EO—EC.

(1) T=diag(a)(C−O)

Then we performed feature selection on **T** based on the **F**_*peak*_, **F**_*mean*_ and **F**_*all*_ these three feature selection methods. After that, we got a new matrix **X** which would be the input matrix into the SVM classifier.

(2) X=diag(a)(C(F)−O(F))

The length of **F**, i.e., **F**_*peak*_, **F**_*mean*_ and **F**_*all*_ is k, k, and 19 × *k* corresponding to Peak-ALFF, Mean-ALFF and All-ALFF feature extraction methods. So the size of **X** is *m* × *k*, *m* × *k* and *m* × (19×*k*). Thus, we obtained 15 samples of EC—EO and 16 samples of EO—EC in dataset-1. Similarly, in dataset-2, we obtained 23 samples of EC—EO and 23 samples of EO—EC. The samples in datasets were labeled with category labels (−1 corresponding to EC—EO group and 1 corresponding to EO—EC group). Previous classification studies on EC and EO have considered the two conditions as independent classes (Liang et al., 2014; Zhang et al., 2015; i.e., the UNPAIR method in the current study). To compare the PAIR and UNPAIR method, we also performed classification between EC and EO while taking them as two independent classes, as described in Liang et al. (2014). The features were also from the voxels (i.e., Peak-ALFF, Mean-ALFF and All-ALFF, respectively), as mentioned above. Therefore, we had 31 EC samples and 31 EO samples for dataset-1, and 46 EC samples and 46 EO samples for dataset-2. The samples in each dataset were labeled with category labels (-1 corresponding to the EC condition and 1 corresponding to the EO condition).

### 2.7. Classification and validation within dataset

Within dataset-1 and dataset-2, we used leave-one-out cross validation (LOOCV). Specifically, for each run of classification, one sample was treated as testing data and the remaining samples were used to construct the training data to perform classification. We obtained the accuracy (0 for wrong and 1 for correct) for each run. All samples went through this process, and the averaged accuracy was obtained. The SVM method is a mathematical programming approach based on the nonlinear optimization problem (Cortes and Vapnik, 1995). SVM has become an increasingly popular method in many fields in recent years (Misaki et al., 2010; Greene et al., 2016; Linn et al., 2016). SVM incorporates the concept of structural risk minimization by creating a separating hyperplane that not only maximizes the margin separating two classes of data, but also minimizes the misclassification error. Suppose we have empirical data ${\left\{X_{i},y_{i}\right\}}_{i=1}^{n}$ with input feature pattern *x*_*i*_ ∈ *X* and binary labels *y*_*i*_ ∈ {+1, −1}, SVM will find a linear hyperplane *h*(*X*) = *W*^*T*^ + *b* that separates the positive from the negative samples with the largest soft margin. To construct this optimal hyperplane, the following mathematical problem must be solved:

(3) $$\begin{array}{l} \;\underset{W,b,\xi }{\min}\frac{1}{2}{\left\|W\right\|}_{2}^{2}+\frac{C}{2}\Sigma_{i=1}^{n}\xi_{i}^{2}\\ s.t.\;y_{i}(W^{T}X_{i}+b)+\xi_{i}\geq 1\;i=1,\ldots ,n \end{array}$$

where ξ_*i*_ are slack variables for the soft margin and *C* is a hyper-parameter between the margin space and the prediction error. In the current study, the LIBLINEAR toolbox (Fan et al., 2008) with the linear support vector classification (SVC) as the classifier for the multi-voxel pattern analysis. *C* is set to 1 here. The SVC classifier was trained on the training data and the corresponding category labels, and the performance of the trained classifier was tested using the testing data and the matching category labels. The LOOCV was performed separately in five frequency bands with three feature extraction methods as mentioned above, using both the PAIR method and the UNPAIR method.

### 2.8. Validation across datasets

**Figure 1:**
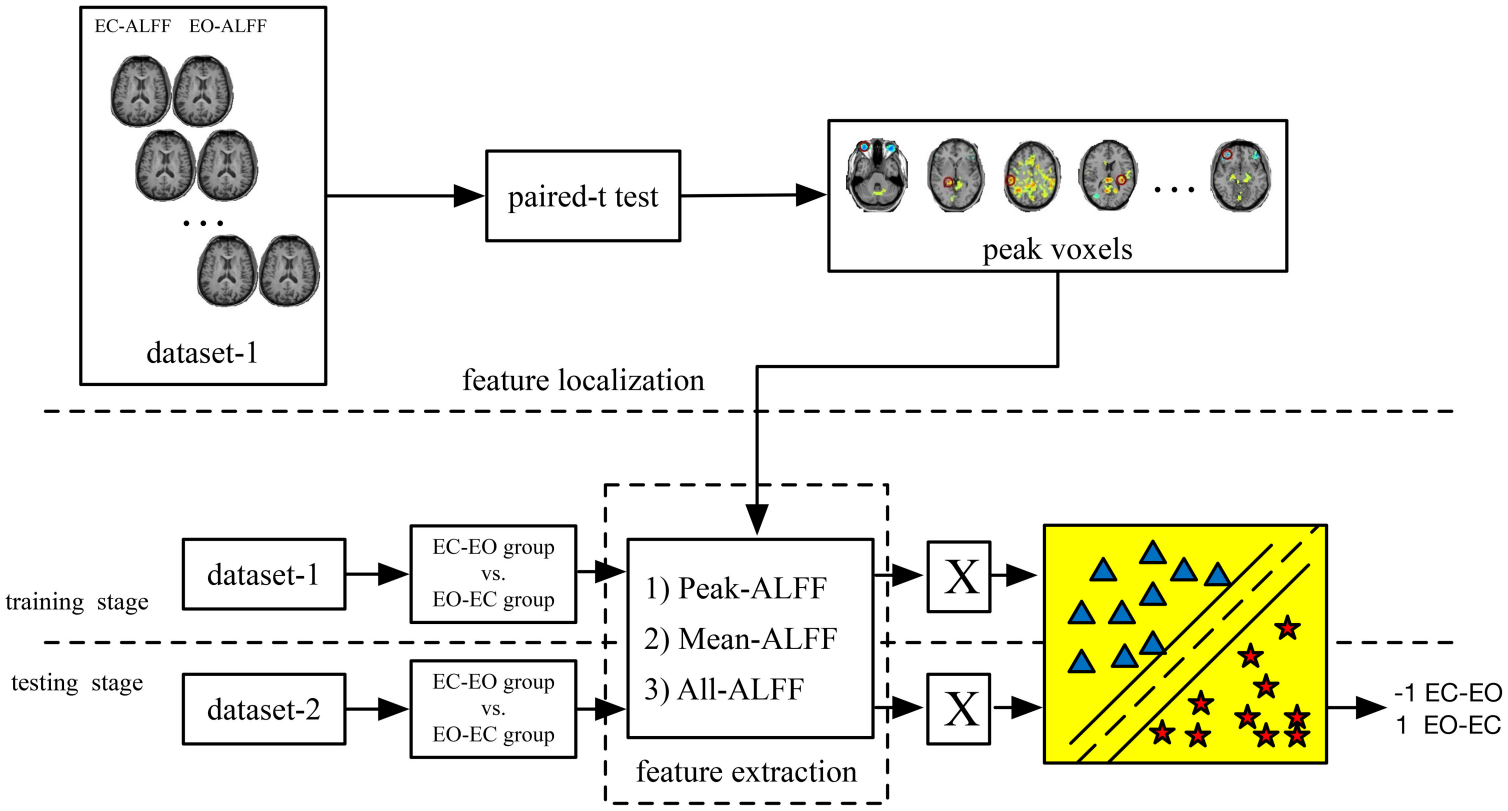
The main steps of the PAIR method (i.e., EC—EO vs. EO–EC groups). The classification was performed separately on Peak-ALFF (ALFF of the peak voxel), Mean-ALFF (mean ALFF of a spherical ROI) and All-ALFF (ALFF of all voxels of a spherical ROI). **X** is the input matrix into the classifier).

**Figure 2:**
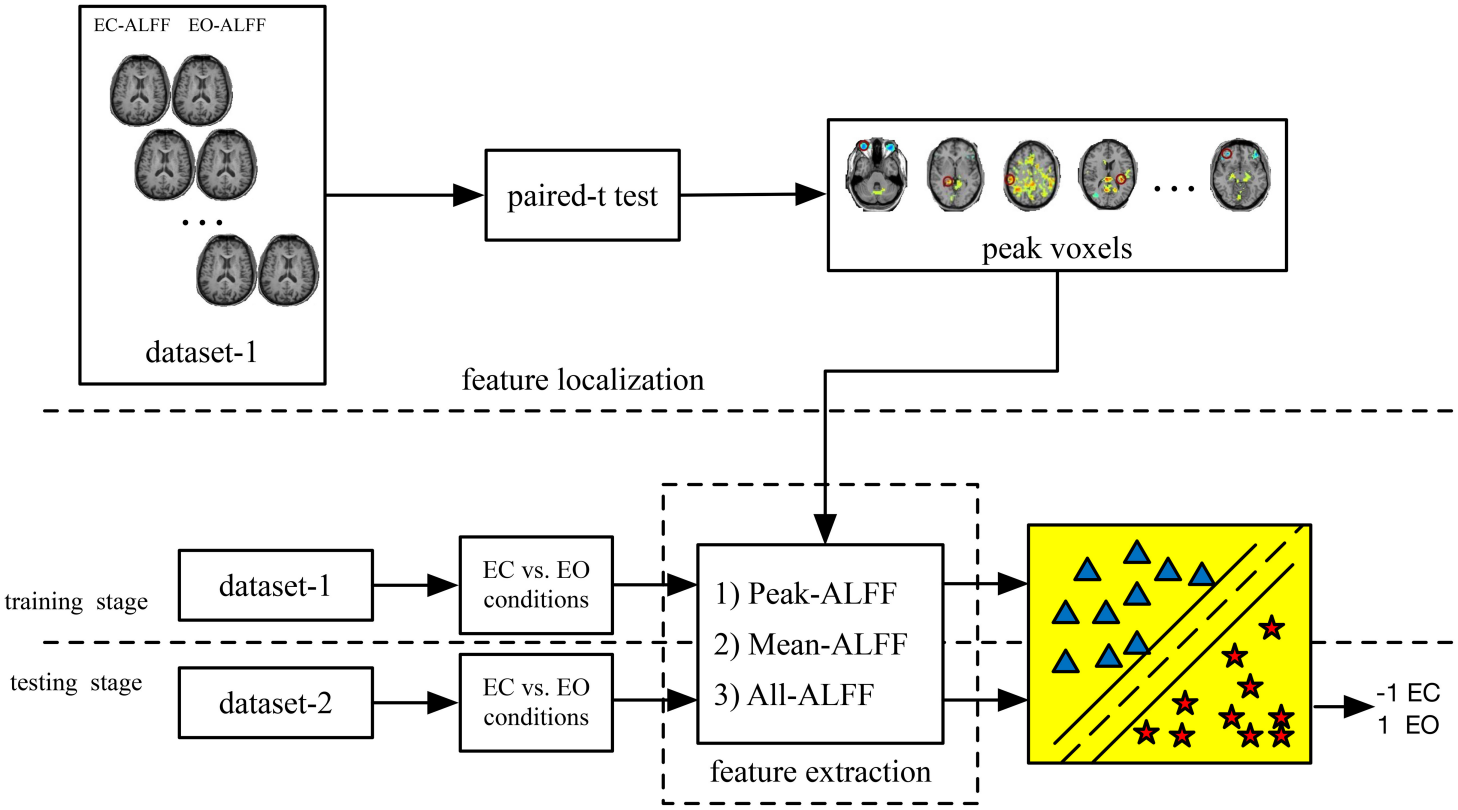
The main steps of the UNPAIR method (i.e., EC vs. EO conditions). Classification was performed separately on Peak-ALFF (ALFF of the peak voxel), Mean-ALFF (mean ALFF of a spherical ROI) and All-ALFF (ALFF of all voxels of a spherical ROI). Unlike the method shown in Figure 1, this method directly differentiated between EC and EO conditions.

To determine whether the features and corresponding classifiers in one dataset could be generalized to the other dataset using the current method, we performed cross-dataset validation. Thus, the classifier obtained in dataset-1 was directly tested on dataset-2, and vice versa. The whole process is shown in Figure 1. In addition, we reversed the whole experimental procedure, as shown in Figure 2 (i.e., training on dataset-2 and testing on dataset-1).

## 3. Results

### 3.1. Paired *t*-test results

**Figure 3:**
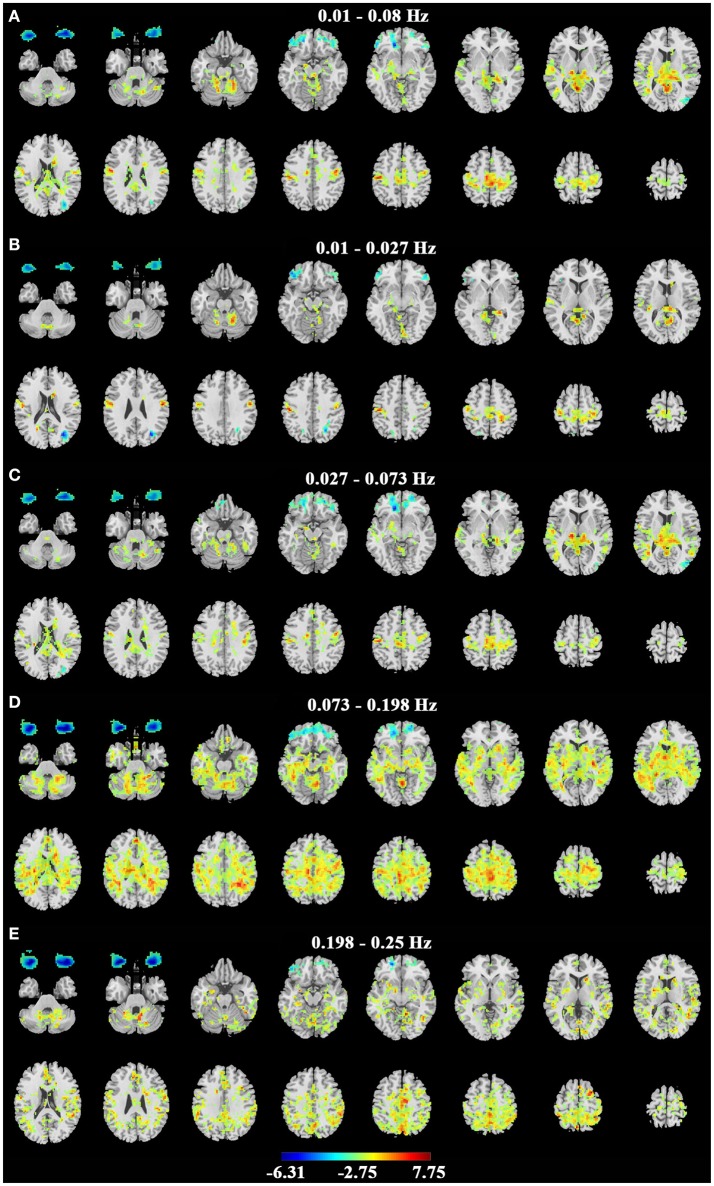
Paired *t*-test results of ALFF on dataset-1. **(A–E)** represent the difference of ALFF between EC and EO in the conventional band (0.01–0.08 Hz), Slow-5 (0.01–0.027 Hz), Slow-4 (0.027–0.073 Hz), Slow-3 (0.073–0.198 Hz) and Slow-2 (0.198–0.25 Hz) bands. Left in the figure indicates the right side of the brain. Warm colors indicate higher ALFF in EC than EO, and cold colors indicate the opposite. (*p* < 0.01, corrected).

**Figure 4:**
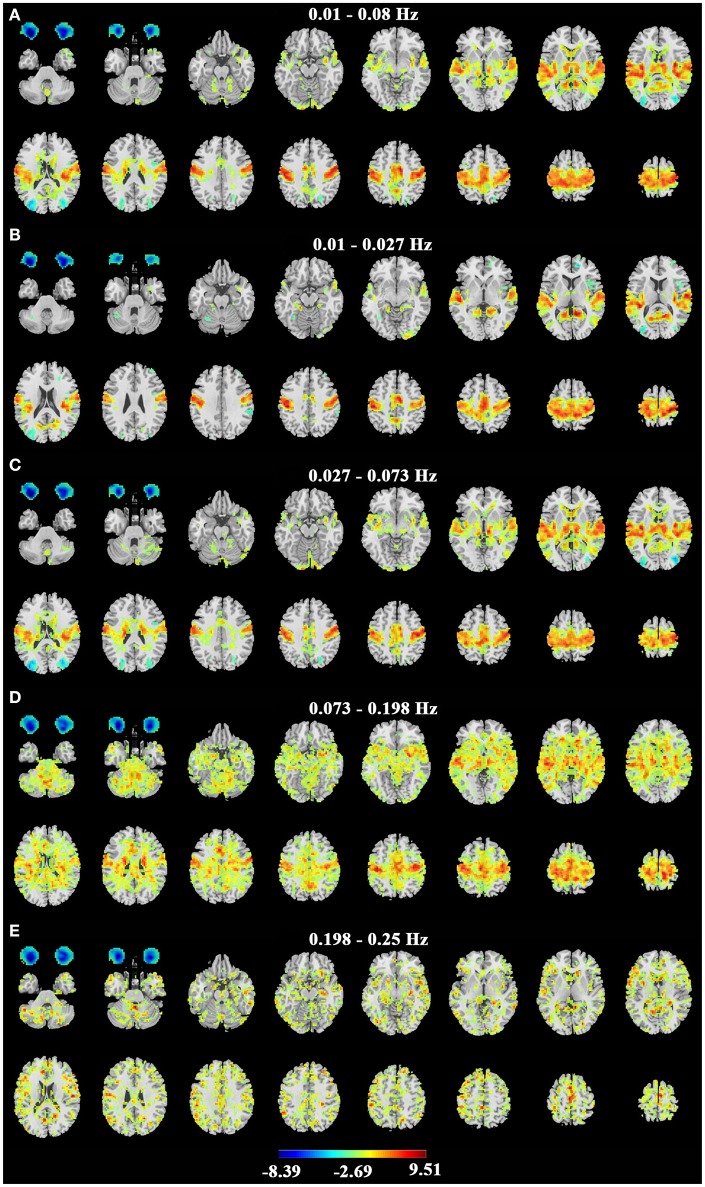
Paired *t*-test results of ALFF on dataset-2. **(A–E)** represent the difference of ALFF between EC and EO in the conventional band (0.01–0.08 Hz), Slow-5 (0.01–0.027 Hz), Slow-4 (0.027–0.073 Hz), Slow-3 (0.073–0.198 Hz) and Slow-2 (0.198–0.25 Hz) bands. Left in the figure indicates the right side of the brain. Warm colors indicate higher ALFF in EC than EO, and cold colors indicate the opposite. (*p* < 0.01, corrected).

Consistent with previous studies (Liu et al., 2013; Qin et al., 2013; Xu et al., 2014; Yuan et al., 2014; Zou et al., 2015), the EC condition exhibited significantly higher ALFF in the primary sensorimotor cortex, the primary auditory cortex, the thalamus and some parietal regions (Figure 3 for dataset-1 and Figure 4 for dataset-2). The EO condition showed significantly higher ALFF in the eye ball, the frontal pole and the lateral occipital area. The results showed some frequency-dependent differences. For example, the higher frequency bands of Slow-2 (0.198–0.25 Hz) and Slow-3 (0.073–0.198 Hz) showed some differences in the white matter and eyeballs. The difference pattern was similar for the two datasets. Detailed information about the coordinates of the peak voxels is shown in Supplementary Tables 1, 2.

### 3.2. SVM classifier performance

We tested the classification performance of both the PAIR method and the UNPAIR method both within each dataset (by LOOCV) and across datasets. As shown in Table 1, in the conventional frequency band of 0.01–0.08 Hz, the classification accuracy of the PAIR method within dataset-1 was 91.40%, and was 92.75% within dataset-2. The mean accuracy of cross-dataset validation was 94.93% for dataset-1 to dataset-2 and 90.32% for dataset-2 to dataset-1 in the 0.01–0.08 Hz (Table 1, in bold font). This finding indicates that classification accuracy with the PAIR method was better than that of the UNPAIR method. The classification accuracy of the three feature selection methods in five frequency bands are listed in Table 1.

**Table 1:** Comparison of classification accuracy between PAIR and UNPAIR methods.

	**Frequency band**	**0.01–0.08 Hz**	**0.01–0.027 Hz**	**0.027–0.073 Hz**	**0.073–0.198 HZ**	**0.198–0.25 Hz**
Peak-ALFF	LOOCV on dataset-1	93.55%	100%	90.32%	100%	93.55%
Mean-ALFF	LOOCV on dataset-1	93.55%	100%	93.55%	96.77%	93.55%
All-ALFF	LOOCV on dataset-1	87.10%	100%	96.77%	100%	90.32%
Mean accuracy		**91.40%**	100%	93.55%	98.92%	92.47%
Peak-ALFF	LOOCV on dataset-2	93.48%	93.48%	95.65%	95.65%	93.48%
Mean-ALFF	LOOCV on dataset-2	95.65%	93.48%	95.65%	95.65%	93.48%
All-ALFF	LOOCV on dataset-2	89.13%	84.78%	89.13%	91.30%	91.30%
Mean accuracy		**92.75%**	90.58%	93.48%	94.20%	92.75%
Peak-ALFF	dataset-1 to dataset-2	93.48%	84.78%	84.78%	95.65%	95.65%
Mean-ALFF	dataset-1 to dataset-2	95.65%	84.78%	91.30%	95.65%	95.65%
All-ALFF	dataset-1 to dataset-2	95.65%	84.78%	93.48%	89.13%	95.65%
Mean accuracy		**94.93%**	84.87%	89.85%	93.48%	95.65%
Peak-ALFF	dataset-2 to dataset-1	83.87%	83.87%	90.32%	93.55%	87.10%
Mean-ALFF	dataset-2 to dataset-1	93.55%	80.65%	93.55%	93.55%	93.55%
All-ALFF	dataset-2 to dataset-1	93.55%	93.55%	90.32%	83.87%	100%
Mean accuracy		**90.32%**	86.02%	91.40%	90.32%	93.55%
**EC—EO vs. EO—EC GROUPS**						
Peak-ALFF	LOOCV on dataset-1	75.81%	77.42%	72.58%	80.65%	79.03%
Mean-ALFF	LOOCV on dataset-1	77.42%	74.19%	74.19%	82.26%	77.42%
All-ALFF	LOOCV on dataset-1	56.45%	69.35%	66.13%	72.58%	67.74%
Mean accuracy		**69.89%**	73.65%	70.97%	78.50%	75.73%
Peak-ALFF	LOOCV on dataset-2	83.70%	85.87%	85.87%	89.13%	92.39%
Mean-ALFF	LOOCV on dataset-2	83.70%	85.87%	85.87%	91.30%	90.22%
All-ALFF	LOOCV on dataset-2	81.52%	89.13%	82.61%	82.61%	80.43%
Mean accuracy		**82.97%**	86.96%	84.78%	87.68%	87.68%
Peak-ALFF	dataset-1 to dataset-2	60.87%	63.04%	68.48%	71.74%	67.39%
Mean-ALFF	dataset-1 to dataset-2	69.57%	64.13%	79.35%	71.74%	70.65%
All-ALFF	dataset-1 to dataset-2	63.04%	75%	75%	73.91%	85.87%
Mean accuracy		**64.49%**	67.39%	74.28%	72.46%	74.64%
Peak-ALFF	dataset-2 to dataset-1	61.29%	56.45%	66.13%	72.58%	74.19%
Mean-ALFF	dataset-2 to dataset-1	64.52%	58.06%	62.90%	69.35%	77.42%
All-ALFF	dataset-2 to dataset-1	66.13%	64.52%	59.58%	74.19%	75.81%
Mean accuracy		**63.98%**	59.68%	62.87%	72.04%	75.81%
**EC vs. EO CONDITIONS**						

**Figure 5:**
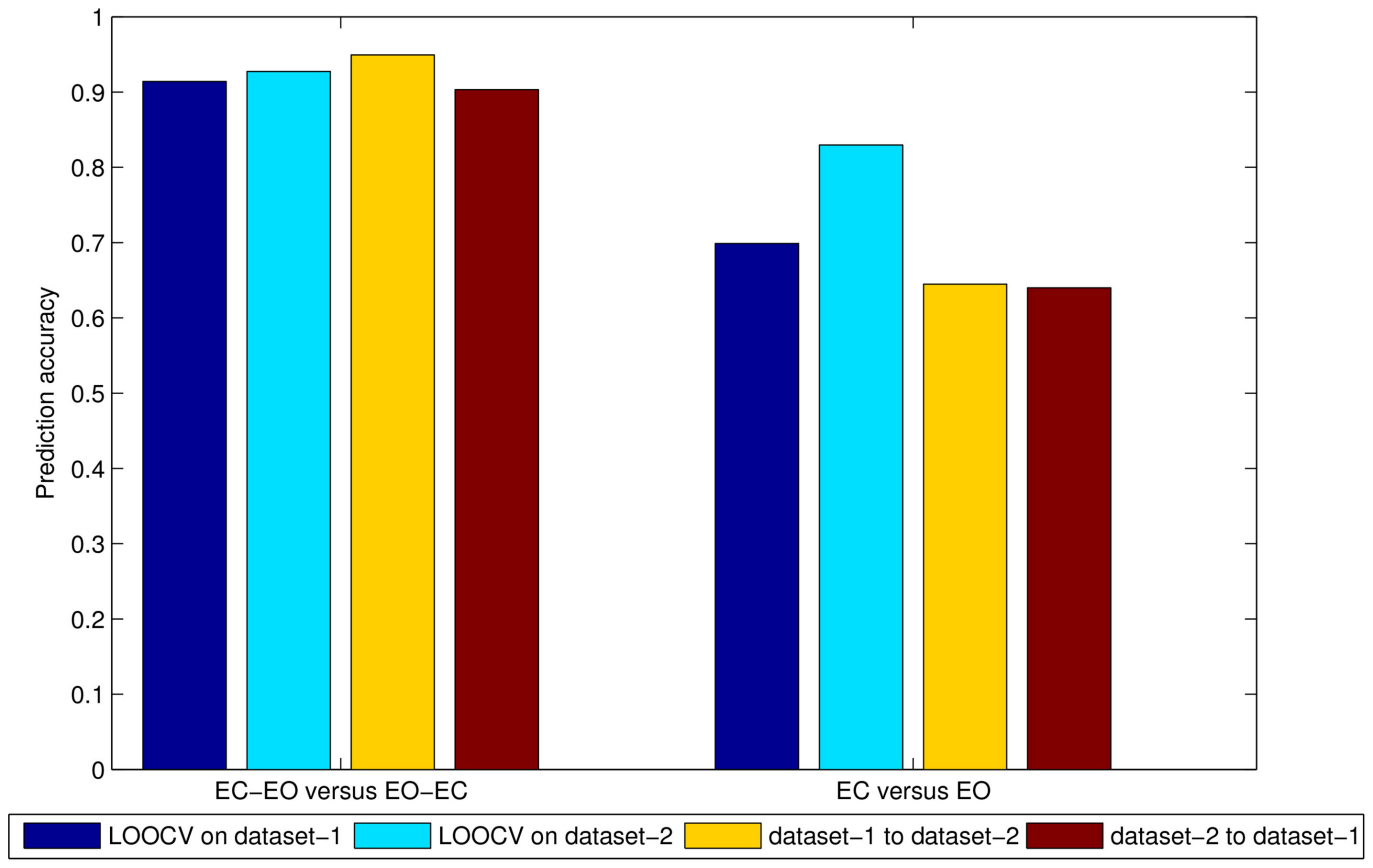
Mean accuracy of three feature selection methods in the 0.01–0.08 Hz band in both the PAIR and UNPAIR methods. The left side means EC—EO vs. EO—EC groups, while the right side denotes EC vs. EO conditions. The dark blue bar shows the accuracy of LOOCV on dataset-1, the light blue bar shows the accuracy of LOOCV on dataset-2, the orange bar shows the accuracy of the classifier of cross-dataset validation from dataset-1 to dataset-2, and the dark red bar shows the accuracy of the classifier of cross-dataset validation from dataset-2 to dataset-1.

To directly compare the PAIR method and UNPAIR method, we averaged the performance of three feature selection methods in conventional frequency band of 0.01–0.08 Hz as shown in Figure 5. With the PAIR method, the classification accuracy within dataset-1 was 91.40%, and accuracy was 92.75% within dataset-2. The mean accuracy of cross-dataset validation was 94.93% for dataset-1 to dataset-2 and 90.32% for dataset-2 to dataset-1. With the UNPAIR method, the classification accuracy within dataset-1 was around 69.89% and accuracy was 82.97% within dataset-2. The mean accuracy of cross-dataset validation was 64.49% for dataset-1 to dataset-2 and 63.98% for dataset-2 to dataset-1. Thus, there was a clear difference in classification accuracy between the two methods.

**Figure 6:**
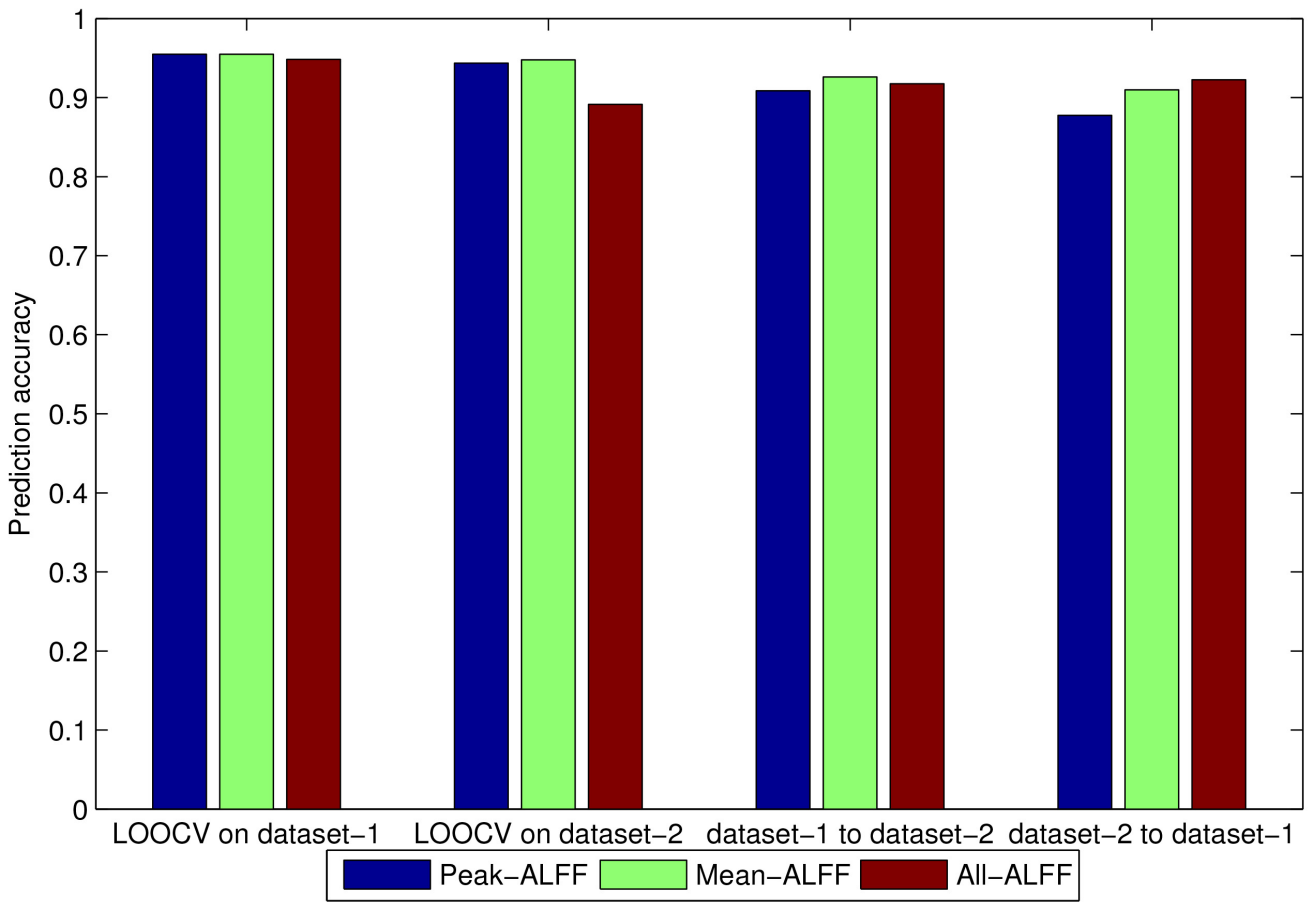
Comparison among three feature selection methods, i.e., Peak-ALFF, Mean-ALFF, and All-ALFF in PAIR method (i.e., EC—EO group vs. EO—EC group). The five frequency bands were averaged.

We also compared the three feature selection methods (i.e., Peak-ALFF, Mean-ALFF, and All-ALFF) by averaging the accuracy of five frequency bands for each feature selection method. Since the classification accuracy of UNPAIR method was worse than that of the PAIR method, the comparison among feature selection methods was only conducted for the PAIR method. As shown in Figure 6, the classification accuracy was similar for the three feature selection methods, while Mean-ALFF was slightly more stable than the other methods. Thus, further analysis was based on Mean-ALFF.

**Figure 7:**
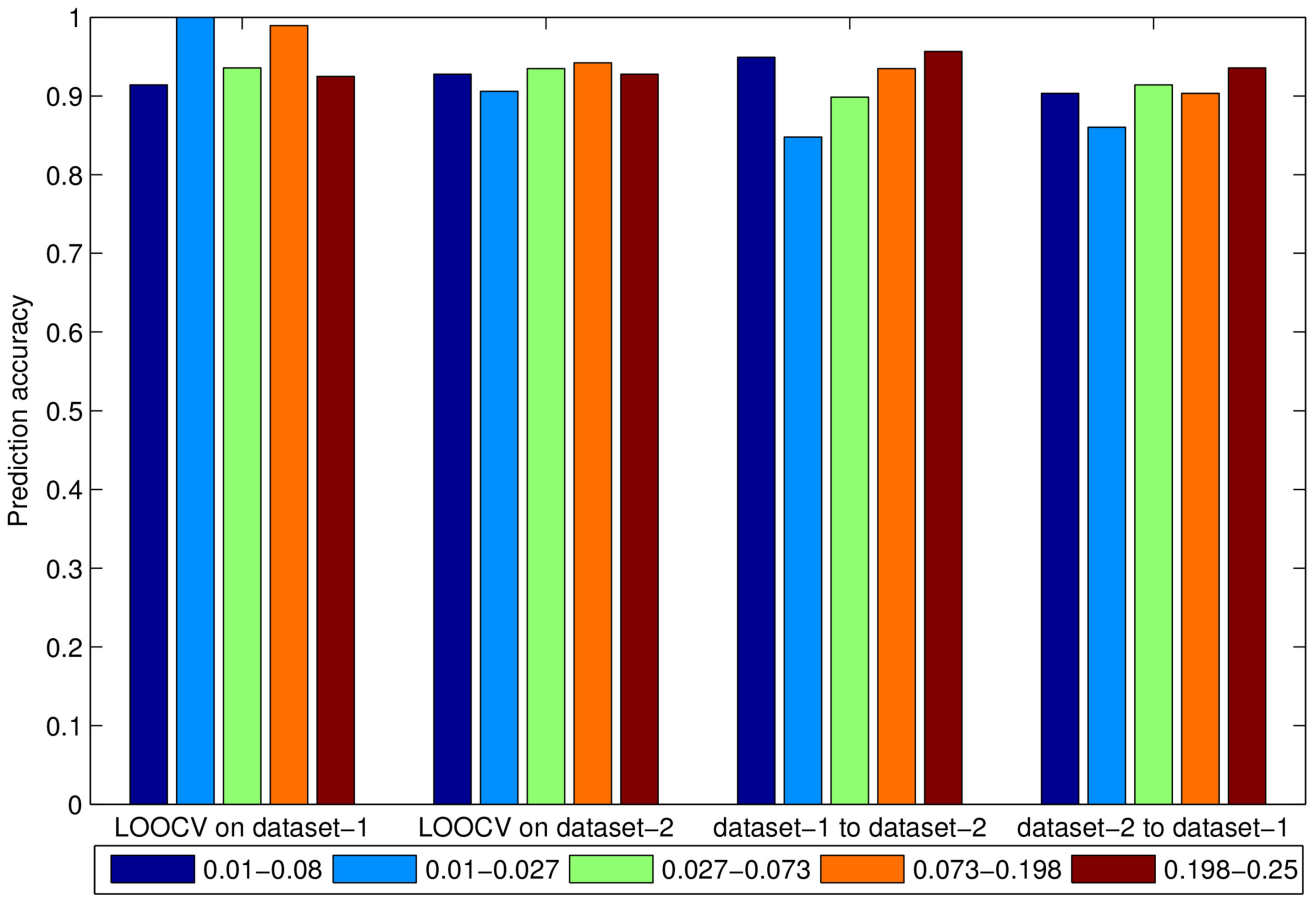
Accuracy of the averaged three feature selection methods in 0.01–0.08 Hz, 0.01–0.027 Hz, 0.027–0.073 Hz, 0.073–0.198 Hz, and 0.198–0.25 Hz bands in the PAIR method.

Next we compared the classification accuracy of the PAIR method among five frequency bands. We averaged the accuracy of three feature selection methods. As shown in Figure 7, in most conditions, the 0.073–0.198 Hz and 0.198–0.25 Hz bands resulted in better prediction accuracy. The very low frequency band of 0.01–0.027 Hz resulted in lower accuracy in the cross-dataset validations of both dataset-1 to dataset-2 and dataset-2 to dataset-1.

### 3.3. Weight of features

One advantage of linear SVM is that the weights of all features (i.e., brain regions in the current study) for classification accuracy can be obtained. *W* in Equation (3) is the weight of each feature, determining the importance of the contribution to this feature to the classification. The greater the absolute value, the more important it is. We analyzed the feature weights of the classifier with LOOCV within each dataset and via cross-dataset validation in five frequency bands using the PAIR method by Mean-ALFF features. The results of the conventional frequency band (0.01–0.08 Hz) are shown in Table 2. The other results are listed in Supplementary Tables 3, 4.

**Table 2:** Weight of each brain region of LOOCV in dataset-1 and dataset-2, and by cross-validation between datasets in the 0.01–0.08 Hz band.

**Brain regions of dataset-1 0.01–0.08 Hz**	**Cluster index**	**ROI mean *t*-value**	**Weight of features by LOOCV in dataset-1 (93.55%)**	**Weight of features by across validation from dataset-1 to dataset-2 (95.65%)**
Eyeball, R	1	−4.7459	0.0885	0.0600
SMC, B	2	5.2508	−1.0305	−1.0665
Eyeball, L	3	−4.7839	0.2405	0.2436
Frontal_Inf_Orb, R	4	−3.2028	0.3005	0.3111
Frontal_Inf_Orb, L	5	−4.1089	0.4917	0.5112
Occipital_Mid, R	6	−4.0079	1.4032	1.4368
Caudate, R	7	4.1222	−0.4066	−0.4168
Cingulum_Mid, R	8	3.1957	0.1112	0.1258
**Brain regions of dataset-2 0.01–0.08 Hz**	**Cluster index**	**ROI mean** ***t*****-value**	**Weight of features by LOOCV in dataset-2 (95.65%)**	**Weight of features by across validation from dataset-2 to dataset-1 (93.55%)**
Cerebelum_8, L	1	2.6758	−0.2981	−0.3097
Eyeball, R	2	−7.0039	0.1833	0.1897
Eyeball, L	3	−7.6689	0.0135	0.0132
Lingual, L	4	3.1218	−0.7927	−0.8088
Cerebelum_Crusl, R	5	3.2954	−0.5689	−0.5715
Cerebelum_4_5, R	6	3.2602	−0.4425	−0.4543
SMC, B	7	6.8304	−0.7927	−0.7956
Occipital_Mid, L	8	−0.4900	0.2756	0.2758

SMC, sensorimotor cortex; Inf, inferior; Mid, middle;

*Orb, orbital; L, left; R, right; B, bilateral*.

As shown in Table 2, the *t*-values were correlated with the weights. However, considering the small sample size of brain regions (*n* = 8 for each dataset), we combined the two datasets and found a significant linear correlation between the *t*-values and the weight (*r* = −0.7242, *p* < 0.05, *n* = 16; here we discussed the weights in LOOCV). It should be noted that the negative correlation was due to the arbitrary labeling of datasets (−1 and 1).

Table 2 shows that the right eyeball (cluster number 1) in dataset-1 had a large absolute *t*-value (−4.7459) but a low weight (0.0885). In dataset-2, the left eyeball (cluster number 3) showed a similar pattern (*t* = −7.6689, weight = 0.0135). Thus, we eliminated the right eyeball in dataset-1 and eliminated the left eyeball in dataset-2, and repeated the classification. Comparing the results in Table 3 with those in Table 2, the classification accuracy after removing the right eyeball in dataset-1 and removing the left eyeball in dataset-2 remained roughly the same. The absolute value of the linear correlation coefficient (combining dataset-1 and dataset-2, *n* = 14 after removing) between the *t*-value and the new weight increased slightly (*r* = −0.7242 before elimination, but -0.7772 after elimination). This finding indicates that although we removed certain abnormal brain regions for dataset-1 and dataset-2, the classification accuracy remained similar. The relationship between the *t*-values and weight in SVM was more complex, and requires in-depth investigation in future studies.

**Table 3:** Weight of each brain region of LOOCV in dataset-1 and dataset-2, and by cross-validation between datasets in the 0.01–0.08 Hz band (removing features with the lowest absolute weight).

**Brain regions of dataset-1 0.01–0.08 Hz**	**Cluster index**	**ROI mean *t*-value**	**Weight of features by LOOCV in dataset-1 (96.77%)**	**Weight of features by across validation from dataset-1 to dataset-2 (95.65%)**
SMC, B	2	5.2508	−1.0516	−1.0851
Eyeball, L	3	−4.7839	0.3373	0.3374
Frontal_Inf_Orb, R	4	−3.2028	0.3499	0.3610
Frontal_Inf_Orb, L	5	−4.1089	0.5260	0.5439
Occipital_Mid, R	6	−4.0079	1.2911	1.3110
Caudate, R	7	4.1222	−0.4244	−0.4327
Cingulum_Mid, R	8	3.1957	0.1815	0.1962
**Brain regions of dataset-2 0.01–0.08 Hz**	**Cluster index**	**ROI mean** ***t*****-value**	**Weight of features by LOOCV in dataset-2 (95.65%)**	**Weight of features by across validation from dataset-2 to dataset-1 (93.55%)**
Cerebelum_8, L	1	2.6758	−0.2988	−0.3062
Eyeball, R	2	−7.0039	0.1966	0.2009
Lingual, L	4	3.1218	−0.7958	−0.8045
Cerebelum_Crusl, R	5	3.2954	−0.5693	−0.5777
Cerebelum_4_5, R	6	3.2602	−0.4308	−0.4416
SMC, B	7	6.8304	−0.8045	−0.8160
Occipital_Mid, L	8	−0.4900	0.2768	0.2771

SMC, sensorimotor cortex; Inf, inferior; Mid, middle;

*Orb, orbital; L, left; R, right; B, bilateral*.

As shown in Table 3, the contribution of each brain region was similar for within-dataset validation and for cross-dataset validation. Although the exact location of the peak voxels was different for the two datasets, the sensorimotor cortex (SMC) and visual cortex contributed more than the other regions to the final classification accuracy in both datasets.

## 4. Discussion

Most previous studies examining this topic have used between-group designs (e.g., patients vs. controls), and independent two-sample *t*-test is a popular univariate statistical method. However, within-group designs can reduce between-subject variance and increase statistical power, and the conventional corresponding univariate statistical analysis is the paired *t*-test method. SVM is a popular multivariate statistical method and has been widely used to differentiate between independent groups. However, for within-group designs, to the best of our knowledge, few classification studies have taken paired conditions into account. An important contribution of the current study is that, unlike previous studies that considered EC and EO as independent conditions (i.e., the UNPAIR method), we utilized a within-group design (i.e., the PAIR method) for feature extraction. In the PAIR method, the classification accuracy was relatively high for both within-dataset validation (mean accuracy of 91.40% for dataset-1 and 92.75% for dataset-2) and cross-dataset validation (mean accuracy of 94.93% from dataset-1 to dataset-2 and 90.32% from dataset-2 to dataset-1). However, for the UNPAIR method, the classification accuracy was slightly lower (mean 69.89% for dataset-1 and 82.97% for dataset-2), and was much lower for within-dataset validation (mean accuracy of 64.49% from dataset-1 to dataset-2 and 63.98% from dataset-2 to dataset-1). One possible reason for the better results of the current PAIR method is that with-group design can reduce variability across subjects. The current PAIR classification method can be further applied to other within-group neuroimaging studies, e.g., between two task conditions of task fMRI studies or follow-up studies. Classification of EC and EO is essentially a kind of decoding of brain activities, which will be much helpful to build cyborg intelligent systems (Wu et al., 2013, 2016).

It should be noted that there are a lot of data preprocessing steps for RS-fMRI. One of them is scrubbing to reduce the effect of outliers of timepoints. We thus compared the classification accuracy between scrubbing and without-scrubbing in frequency bands of 0.01–0.08, 0.01–0.027, 0.027–0.073, 0.073–0.198, and 0.198–0.25 Hz in dataset-1. Chi-square tests showed no significant difference (*p* = 0.3134–1 in 5 frequency bands, see Supplementary Material) between the results with scrubbing and without scrubbing.

Many methods have been applied for dimensionality reduction in previous neuroimaging classification studies, and *t*-tests are one of the most widely used methods. However, few studies have investigated the correlation of *t*-values of each region or voxel with the weight of the feature in the classifier. As shown in Tables 2, 3, the *t*-values were highly correlated with the weight in the classifier. The current results confirmed the rationale for using *t*-tests for dimensionality reduction. It should be noted that using *t*-tests for dimensionality reduction in the same dataset is a type of circular analysis (Kriegeskorte et al., 2009). Therefore, this method should be used cautiously, and performing validation with independent datasets may provide a more appropriate approach.

Although most previous RS-fMRI studies have focused on the conventional frequency band of 0.01–0.08 Hz, its sub-frequency bands (e.g., 0.01–0.027 Hz and 0.027–0.073 Hz) (Zuo et al., 2010), as well as higher frequency bands (0.073–0.198 Hz and 0.198–0.25 Hz) have drawn increasing attention in recent years (Yuan et al., 2014; Wang et al., 2015). Classification has been widely used in RS-fMRI studies (Yang et al., 2012). However, few classification studies have examined sub-frequency bands. As shown in Table 1, the current study found that higher frequency bands (0.073–0.198 Hz and 0.198–0.25 Hz) had relatively higher accuracy than lower frequency bands in both dataset-1 and dataset-2. It should be noted that the low frequency band of 0.01–0.027 Hz showed consistently lower accuracy on different datasets (mean accuracy of 100% for dataset-1, 90.58% for dataset-2, 84.87% from dataset-1 to dataset-2, and 86.02% from dataset-2 to dataset-1). We included the eyeballs because eye blink is a type of high frequency movement. We predicted that the high frequency amplitude of the RS-fMRI signal in the eyeballs would contribute more to accuracy. As we expected, the eyeballs did exhibit relatively greater weights than other brain regions in the higher frequency bands (0.073–0.198 Hz and 0.198–0.25 Hz) (Supplementary Tables 3, 4). As a contrast, for the conventional frequency band (0.01–0.08 Hz) as well as it sub-bands (0.01–0.027 Hz and 0.027–0.073 Hz), the primary sensorimotor cortex and visual cortex had the largest weights. All of the above results were consistent for dataset-1 and dataset-2.

Feature selection is a minor issue in the current study. Based on *t* maps, we compared three methods for feature selection: peak voxel ALFF (Peak-ALFF), mean ALFF of a spherical ROI (Mean-ALFF) and ALFF of all voxels of a spherical ROI (All-ALFF). The accuracy of the three methods was similar. Because the mean value of a spherical ROI may vary less than in different validations than the peak voxel, and using the ALFF of all voxels in a spherical ROI as features increases the dimensionality, we recommend the Mean-ALFF selection method.

The current study involved several limitations that should be considered. First, to meet the demands of labels used for classification, we randomly divided the data into two groups (i.e., EC—EO group and EO—EC group). However, although we supposed that different grouping methods would not affect the results, we did not provide theoretical evidence. Considering that this type of within-group design is also widely used, future studies should investigate a mathematical solution. Second, although there are many methods for dimensionality reduction, we only used *t*-tests in the current study. Future studies should test other dimensionality reduction methods, which might increase accuracy and consistency. Third, the classification accuracy of the low frequency band of 0.01–0.027 Hz appeared to have low consistency in cross-dataset validation. However, this result is difficult to interpret. Future studies should examine this frequency band of the RS-fMRI signal in more detail.

## Author contributions

ZZ and J-BW contributed equally to this research. Y-FZ and GP conceived and designed the experiment. ZZ and J-BW performed the data analysis. Y-FZ, GP, and J-BW provided advice on the analysis and interpretation of the final results. ZZ wrote the paper.

### Conflict of interest statement

The authors declare that the research was conducted in the absence of any commercial or financial relationships that could be construed as a potential conflict of interest.
