# Supplementary material for: PAIR Comparison between Two Within-Group Conditions of Resting-State fMRI Improves Classification Accuracy
Source: Front Neurosci. 2018 Jan 9;11:740. doi: 10.3389/fnins.2017.00740 (PMC5767225; doi:10.3389/fnins.2017.00740)
Supplement: Supplementary file 1 [file Table1.DOCX]

Supplementary table 1 Brain regions showing statistically significant group differences in dataset-1

| Brain region | BA | MNI (X, Y, Z ) | | | Peak t value |
| --- | --- | --- | --- | --- | --- |
| 0.01-0.08 Hz |  |  |  |  |  |
| Eyeball, R | N/A | 36 | 57 | -36 | -5.2824 |
| SMC, B | 2 | -48 | -21 | 48 | 7.7521 |
| Eyeball, L | N/A | -36 | 54 | -33 | -5.4645 |
| Frontal_Inf_Orb, R | 47 | 42 | 30 | -12 | -4.6265 |
| Frontal_Inf_Orb, L | 11 | -15 | 33 | -9 | -6.3127 |
| Occipital_Mid, R | 19 | 36 | -75 | 18 | -4.7057 |
| Caudate, R | 32 | 15 | 9 | 24 | 5.8641 |
| Cingulum_Mid, R | 32 | 3 | 27 | 36 | 4.6013 |
| 0.01-0.027 Hz |  |  |  |  |  |
| Eyeball, R | N/A | 39 | 60 | -33 | -4.7815 |
| Cerebellum, L, R | 29 | 21 | -39 | 0 | 5.0204 |
| Eyeball, L | N/A | -36 | 54 | -33 | -4.9974 |
| Frontal_Inf_Orb, L | 47 | -42 | 42 | -15 | -5.0278 |
| Frontal_Mid_Orb, R | 47 | 36 | 45 | -12 | -4.8797 |
| Lingual, R | 18 | 3 | -78 | -9 | 4.2269 |
| Rolandic_Oper, L | 13 | -36 | -30 | 15 | 5.3989 |
| Postcentral, R | 6 | 54 | -9 | 27 | 5.9443 |
| Caudate, R | N/A | 15 | 9 | 24 | 5.0922 |
| Occipital_Mid, R | 7 | 33 | -72 | 24 | -6.4965 |
| Calcarine, L | N/A | -21 | -60 | 21 | 4.6552 |
| Parietal_Sup, L | 7 | -21 | -69 | 57 | -3.5055 |
| Postcentral, L | 3 | -45 | -21 | 15 | 5.7228 |
| 0.027-0.073 Hz |  |  |  |  |  |
| Eyeball, L | N/A | -36 | 51 | -51 | -4.4199 |
| Eyeball, R | N/A | 36 | 57 | -36 | -5.4139 |
| Eyeball, L | N/A | -36 | 54 | -33 | -5.3076 |
| Postcentral, L, R | 13 | -27 | -30 | 18 | 7.4052 |
| Frontal Orbital cortex | 32 | -15 | 33 | -9 | -6.7776 |
| Amygdala, L | 28 | -12 | -9 | -15 | 4.3694 |
| Occipital_Mid, R | 19 | 33 | -78 | 18 | -4.5439 |
| Frontal_Sup_Medial, L | 6 | 0 | 27 | 36 | 4.3625 |
| 0.073-0.198 Hz |  |  |  |  |  |
| Eyeball, R | N/A | 39 | 54 | -36 | -6.8501 |
| Eyeball, L | N/A | -36 | 54 | -33 | -7.0976 |
| Putamen, R | 6 | 30 | -18 | 6 | 9.7973 |
| Bilateral orbital frontal cortex | 11 | -15 | 45 | -9 | -5.3854 |
| 0.198-0.25 Hz |  |  |  |  |  |
| Eyeball, L | N/A | -30 | 57 | -30 | -7.8302 |
| Eyeball, R | N/A | 42 | 57 | -36 | -7.1864 |
| SupraMarginal, R | 2 | 63 | -27 | 36 | 6.8514 |
| Frontal_Inf_Orb, L | 47 | -45 | 42 | -15 | -5.087 |
| Frontal_Mid_Orb, R | 11 | 27 | 51 | -12 | -5.3813 |
| Frontal_Sup_Orb, L | 11 | -18 | 51 | -9 | -7.0175 |
| BA = Brodmann area, MNI = Montreal Neurological Institute, L = left hemisphere,  N/A = not available, R = right hemisphere  N/A = not available, R = right hemisphere | | | | | |

Supplementary table 2 Brain regions showing statistically significant group differences in dataset-2

| Brain region | BA | MNI (X, Y, Z ) | | | Peak t value |
| --- | --- | --- | --- | --- | --- |
| 0.01-0.08 Hz |  |  |  |  |  |
| Cerebelum_8, L | N/A | -15 | -57 | -57 | 5.0361 |
| Eyeball, R | N/A | 36 | 60 | -33 | -7.7582 |
| Eyeball, L | N/A | -33 | 57 | -36 | -8.395 |
| Lingual, L | 18 | -12 | -99 | -15 | 6.0773 |
| Cerebelum_Crusl, R | 37 | 45 | -69 | -24 | 5.2663 |
| Cerebelum_4_5, R | 36 | 18 | -54 | -18 | 4.732 |
| Precentral, R | 6 | 57 | -3 | 39 | 9.506 |
| Occipital_Mid, L | 19 | -33 | -78 | 18 | -5.6573 |
| 0.01-0.027 Hz |  |  |  |  |  |
| Eyeball, R | N/A | 36 | 54 | -36 | -7.0306 |
| Eyeball, L | N/A | -39 | 57 | -36 | -7.7737 |
| Cerebelum_8, L | N/A | -33 | -54 | -48 | -5.1881 |
| Cerebelum_Crusl, L | N/A | -39 | -48 | -33 | -3.9266 |
| Lingual, R | 29 | 12 | -51 | 6 | 7.542 |
| Occipital_Inf, R | 18 | 30 | -96 | -12 | 4.7076 |
| Rolandic_Oper, R | 6 | 60 | -9 | 15 | 7.7786 |
| Frontal_Sup_Medial, R | 10 | 15 | 51 | 6 | -4.7866 |
| Thalamus, R | N/A | -6 | -6 | 6 | 3.9778 |
| Frontal_Inf_Oper, R | 13 | 42 | 0 | 24 | -4.5756 |
| Occipital_Mid, L | 19 | -30 | -81 | 15 | -4.3587 |
| Occipital_Mid, R | 19 | 36 | -87 | 27 | -4.063 |
| Frontal_Mid, R | 9 | 36 | 45 | 39 | -3.5561 |
| SupraMarginal, R | 40 | 60 | -39 | 36 | -4.3317 |
| 0.027-0.073 Hz |  |  |  |  |  |
| Vermis_8 | N/A | 6 | -63 | -39 | 5.0857 |
| Cerebelum_6, L | N/A | -24 | -54 | -27 | 4.8517 |
| Eyeball, R | N/A | 36 | 60 | -33 | -7.6912 |
| Eyeball, L | N/A | -33 | 57 | -33 | -8.3602 |
| Heschl, L | 42 | -60 | -9 | 9 | 9.2688 |
| Lingual, L | 18 | -12 | -99 | -15 | 6.4385 |
| Occipital_Mid, R | 19 | 33 | -78 | 12 | -5.6203 |
| Occipital_Mid, L | 19 | -36 | -84 | 21 | -5.9736 |
| 0.073-0.198 Hz |  |  |  |  |  |
| Precentral, R | 4 | 36 | -18 | 51 | 9.7818 |
| Eyeball, R | N/A | 33 | 57 | -30 | -9.3129 |
| Eyeball, L | N/A | -33 | 57 | -33 | -10.2125 |
| 0.198-0.25 Hz |  |  |  |  |  |
| Supp_Motor_Area, R | 6 | 3 | -12 | 66 | 6.8865 |
| Eyeball, R | N/A | 36 | 57 | -33 | -9.8365 |
| Eyeball, L | N/A | -33 | 57 | -33 | -10.761 |
| BA = Brodmann area, MNI = Montreal Neurological Institute, L = left hemisphere,  N/A = not available, R = right hemisphere | | | | | |

Supplementary table 3 Weight of features in classifiers of LOOCV in dataset-1 and by across validation from dataset-1 to dataset-2 in 0.01-0.08 Hz using Mean-ALFF features by the PAIR method

| Brain region | Cluster  index | Weight of features by LOOCV in dataset -1 | Weight of features by across validation from dataset-1 to dataset-2 |
| --- | --- | --- | --- |
| 0.01-0.08 Hz |  |  |  |
| Eyeball | 1 | 0.0885 | 0.0600 |
| Postcentral, L | 2 | -1.0305 | -1.0665 |
| Eyeball | 3 | 0.2405 | 0.2436 |
| Frontal_Inf_Orb, R | 4 | 0.0305 | 0.3111 |
| Frontal_Inf_Orb, L | 5 | 0.4917 | 0.5112 |
| Occipital_Mid, R | 6 | 1.4032 | 1.4368 |
| Caudate, R | 7 | -0.4066 | -0.4168 |
| Cingulum_Mid, R | 8 | 0.1112 | 0.1258 |
| 0.01-0.027 Hz |  |  |  |
| Eyeball | 1 | -0.0517 | -0.0482 |
| Cerebellum, L, R | 2 | -0.1747 | -0.1767 |
| Eyeball | 3 | 0.2918 | 0.2917 |
| Frontal_Inf_Orb, L | 4 | 0.3077 | 0.3129 |
| Frontal_Mid_Orb, R | 5 | -0.0214 | -0.0189 |
| Lingual, R | 6 | -0.7890 | -0.7988 |
| Rolandic_Oper, L | 7 | -0.4893 | -0.4992 |
| Postcentral, R | 8 | -0.5776 | -0.5810 |
| Caudate, R | 9 | -0.3110 | -0.3195 |
| Occipital_Mid, R | 10 | 0.5991 | 0.0600 |
| Calcarine, L | 11 | -0.4792 | -0.4888 |
| Parietal_Sup, L | 12 | 0.1332 | 0.1320 |
| Postcentral, L | 13 | -0.4154 | -0.4238 |
| 0.027-0.073 Hz |  |  |  |
| Eyeball | 1 | 0.2897 | 0.2926 |
| Eyeball | 2 | 0.0734 | 0.0638 |
| Eyeball | 3 | 0.1061 | 0.1039 |
| Postcentral, L, R | 4 | -0.4762 | -0.4911 |
| Frontal Orbital cortex | 5 | 0.5362 | 0.5539 |
| Amygdala, L | 6 | -0.6794 | -0.7085 |
| Occipital_Mid, R | 7 | 1.4101 | 1.4424 |
| Frontal_Sup_Medial, L | 8 | 0.2540 | 0.2676 |
| 0.073-0.198 Hz |  |  |  |
| Eyeball | 1 | 0.2056 | 0.1995 |
| Eyeball | 2 | 0.7773 | 0.7899 |
| Putamen, R | 3 | -0.3478 | -0.3566 |
| Frontal cortex | 4 | 0.3143 | 0.3237 |
| 0.198-0.25 Hz |  |  |  |
| Eyeball | 1 | 0.7187 | 0.7254 |
| Eyeball | 2 | 0.2084 | 0.2289 |
| SupraMarginal, R | 3 | -0.1812 | -0.1884 |
| Frontal_Inf_Orb, L | 4 | 0.2223 | 0.2236 |
| Frontal_Mid_Orb, R | 5 | 0.5886 | 0.5980 |
| Frontal_Sup_Orb, L | 6 | 0.6173 | 0.6327 |

Supplementary table 4 Weight of features in classifiers of LOOCV in dataset-2 and by across validation from dataset-2 to dataset-1 in 0.01-0.08 Hz using Mean-ALFF features by the PAIR method

| Brain region | Cluster  index | Weight of features by LOOCV in dataset -2 | Weight of features by across validation from dataset-2 to dataset-1 |
| --- | --- | --- | --- |
| 0.01-0.08 Hz |  |  |  |
| Cerebelum_8, L | 1 | -0.2981 | -0.3097 |
| Eyeball | 2 | 0.1833 | 0.1897 |
| Eyeball | 3 | 0.0135 | 0.0132 |
| Lingual, L | 4 | -0.7927 | -0.8088 |
| Cerebelum_Crusl, R | 5 | -0.5689 | -0.5715 |
| Cerebelum_4_5, R | 6 | -0.4425 | -0.4543 |
| Precentral, R | 7 | -0.7927 | -0.7956 |
| Occipital_Mid, L | 8 | 0.2756 | 0.2758 |
| 0.01-0.027 Hz |  |  |  |
| Eyeball | 1 | 0.1551 | 0.1500 |
| Eyeball | 2 | 0.0425 | 0.0430 |
| Cerebelum_8, L | 3 | 0.2452 | 0.2502 |
| Cerebelum_Crusl, L | 4 | 0.2922 | 0.2986 |
| Lingual, R | 5 | -0.6852 | -0.6887 |
| Occipital_Inf, R | 6 | -0.1011 | -0.0943 |
| Rolandic_Oper, R | 7 | -0.1368 | -0.1306 |
| Frontal_Sup_Medial, R | 8 | 0.4337 | 0.4479 |
| Thalamus, R | 9 | -0.4175 | -0.4212 |
| Frontal_Inf_Oper, R | 10 | 0.2592 | 0.2667 |
| Occipital_Mid, L | 11 | 0.4305 | 0.4417 |
| Occipital_Mid, R | 12 | 0.3572 | 0.3604 |
| Frontal_Mid, R | 13 | 0.1603 | 0.1556 |
| SupraMarginal, R | 14 | 0.9817 | 0.9924 |
| 0.027-0.073 Hz |  |  |  |
| Vermis_8 | 1 | 0.2398 | 0.2467 |
| Cerebelum_6, L | 2 | -0.0666 | -0.0725 |
| Eyeball | 3 | 0.0374 | 0.0417 |
| Eyeball | 4 | 0.2203 | 0.2262 |
| Heschl, L | 5 | -1.1794 | -0.1907 |
| Lingual, L | 6 | -0.7442 | -0.7523 |
| Occipital_Mid, R | 7 | 0.5481 | 0.5466 |
| Occipital_Mid, L | 8 | 0.3785 | 0.3814 |
| 0.073-0.198 Hz |  |  |  |
| Precentral, R | 1 | -0.2531 | -0.2497 |
| Eyeball | 2 | 0.3235 | 0.3316 |
| Eyeball | 3 | 0.2474 | 0.2525 |
| 0.198-0.25 Hz |  |  |  |
| Supp_Motor_Area, R | 1 | -0.2961 | -0.3060 |
| Eyeball | 2 | 0.1533 | 0.1616 |
| Eyeball | 3 | 0.2731 | 0.2633 |

# Scrubbing

Scrubbing outliers of RS-fMRI data points is a matter of debate for resting-state fMRI data preprocessing. To investigate whether scrubbing will affect the classification performance, we compared the classification accuracy between with- and without-scrubbing in the dataset-1 based on leave-one-out cross-validation. We add the scrubbing procedure in the preprocessing after removing linear trends of the data. We removed time points using a threshold of framewise displacement > 0.2 mm as well as 1 back and 2 forward neighbors (Power et al., 2012). The results of accuracy were shown in Table S5.

Supplementary table 5 Classification accuracy between with- and without- scrubbing in dataset-1 by leave-one-out cross validation.

| Accuracy | 0.01-0.08 Hz | 0.01-0.027  Hz | 0.027-0.073  Hz | 0.073-0.198  Hz | 0.198-00.25  Hz |
| --- | --- | --- | --- | --- | --- |
|  | With scrubbing | | | | |
| Peak-ALFF | 90.32% | 96.77% | 96.77% | 100% | 100% |
| Mean-ALFF | 93.55% | 100% | 96.77% | 96.77% | 100% |
| All-ALFF | 93.55% | 93.54% | 96.77% | 100% | 93.54% |
| Mean accuracy | **92.47%** | **96.77%** | **96.77%** | **98.92%** | **97.85%** |
|  | | | | | |
| Accuracy | 0.01-0.08 Hz | 0.01-0.027  Hz | 0.027-0.073  Hz | 0.073-0.198  Hz | 0.198-00.25  Hz |
|  | Without scrubbing | | | | |
| Peak-ALFF | 93.55% | 100% | 90.32% | 100% | 93.55% |
| Mean-ALFF | 93.55% | 100% | 93.55% | 96.77% | 93.55% |
| All-ALFF | 87.10% | 100% | 96.77% | 100% | 90.32% |
| Mean accuracy | **91.40%** | **100%** | **93.55%** | **98.92%** | **92.47%** |

We performed chi-square tests on accuracy between with- and without-scrubbing. The p value was from 0.3134 to 1 in 5 frequency bands. It shows that scrubbing doesn’t show significant effect on the results of our research.

Supplementary table 6 p values of chi-square tests on on accuracy between with- and without-scrubbing

|  | 0.01-0.08 Hz | | 0.01-0.027 Hz | | 0.027-0.073 Hz | | 0.073-0.198  Hz | | 0.198-0.25 Hz | |
| --- | --- | --- | --- | --- | --- | --- | --- | --- | --- | --- |
| With scrubbing | 28.7 | 2.3 | 30 | 1 | 30 | 1 | 30.7 | 0.3 | 30.3 | 0.7 |
| Without scrubbing | 28.3 | 2.7 | 31 | 0 | 29 | 2 | 30.7 | 0.3 | 28.7 | 2.3 |
| p value | 0.8520 | | 0.3134 | | 0.5540 | | 1 | | 0.3437 | |

References

Power, J.D., Barnes, K.A., Snyder, A.Z., Schlaggar, B.L., Petersen, S.E., 2012. Spurious but systematic correlations in functional connectivity MRI networks arise from subject motion. NeuroImage 59, 2142-2154.
